# Pulmonary Infection with Hypervirulent Mycobacteria Reveals a Crucial Role for the P2X7 Receptor in Aggressive Forms of Tuberculosis

**DOI:** 10.1371/journal.ppat.1004188

**Published:** 2014-07-03

**Authors:** Eduardo P. Amaral, Simone C. M. Ribeiro, Verônica R. Lanes, Fabrício M. Almeida, Marcelle R. M. de Andrade, Caio Cesar Barbosa Bomfim, Érika M. Salles, Karina R. Bortoluci, Robson Coutinho-Silva, Mario H. Hirata, José M. Alvarez, Elena B. Lasunskaia, Maria Regina D'Império-Lima

**Affiliations:** 1 Departamento de Imunologia, Instituto de Ciências Biomédicas (ICB), Universidade de São Paulo (USP), São Paulo, São Paulo, Brazil; 2 Laboratório de Biologia do Reconhecer, Universidade Estadual do Norte Fluminense (UENF), Campos dos Goytacazes, Rio de Janeiro, Brazil; 3 Centro de Terapia Celular e Molecular, Departamento de Ciências Biológicas, Universidade Federal de São Paulo, São Paulo, São Paulo, Brazil; 4 Programa de Imunobiologia, Instituto de Biofísica Carlos Chagas Filho, Universidade Federal do Rio de Janeiro, Rio de Janeiro, Rio de Janeiro, Brazil; 5 Instituto National de Ciência e Tecnologia para Pesquisa Translacional em Saúde e Meio Ambiente da Região Amazônica, Rio de Janeiro, Rio de Janeiro, Brazil; 6 Departamento de Química e Toxicologia Clínica, Faculdade de Ciências Farmacêuticas (FCT), University of São Paulo, São Paulo, São Paulo, Brazil; University of Massachusetts, United States of America

## Abstract

The purinergic P2X7 receptor (P2X7R) is a sensor of extracellular ATP, a damage-associated molecule that is released from necrotic cells and that induces pro-inflammatory cytokine production and cell death. To investigate whether the innate immune response to damage signals could contribute to the development of pulmonary necrotic lesions in severe forms of tuberculosis, disease progression was examined in C57BL/6 and P2X7R^−/−^ mice that were intratracheally infected with highly virulent mycobacterial strains (*Mycobacterium tuberculosis* strain 1471 of the Beijing genotype family and *Mycobacterium bovis* strain MP287/03). The low-dose infection of C57BL/6 mice with bacteria of these strains caused the rapid development of extensive granulomatous pneumonia with necrotic areas, intense bacillus dissemination and anticipated animal death. In contrast, in P2X7R^−/−^ mice, the lung pathology presented with moderate infiltrates of mononuclear leukocytes without visible signs of necrosis; the disease attenuation was accompanied by a delay in mortality. *In vitro*, the hypervirulent mycobacteria grew rapidly inside macrophages and induced death by a P2X7R-dependent mechanism that facilitated the release of bacilli. Furthermore, these bacteria were resistant to the protective mechanisms elicited in macrophages following extracellular ATP stimulation. Based on this study, we propose that the rapid intracellular growth of hypervirulent mycobacteria results in massive macrophage damage. The ATP released by damaged cells engages P2X7R and accelerates the necrotic death of infected macrophages and the release of bacilli. This vicious cycle exacerbates pneumonia and lung necrosis by promoting widespread cell destruction and bacillus dissemination. These findings suggest the use of drugs that have been designed to inhibit the P2X7R as a new therapeutic approach to treat the aggressive forms of tuberculosis.

## Author Summary

Nearly 9 million new cases of tuberculosis and 1.3 million deaths are reported yearly worldwide. Most individuals infected with tubercle bacilli remain asymptomatic; however, some develop active tuberculosis due to the reactivation of latent infections. Progressive primary tuberculosis is an alternative form of the disease that mostly affects children and immunocompromised individuals. Extensive pneumonia, pulmonary necrosis and bacillus dissemination characterize some of the aggressive forms of tuberculosis. To investigate the molecular mechanisms that underlie severe disease progression, we used experimental models of relatively resistant C57BL/6 mice that were infected with highly virulent strains of *Mycobacterium tuberculosis* or *Mycobacterium bovis*. Two hypervirulent strains (*Mtb* strain 1471 and *Mbv* strain MP287/03) induced extensive pulmonary inflammation and necrosis in mice and promoted bacillus dissemination and animal death. We hypothesized that the innate immune response to endogenous damage signals from necrotic cells could aggravate the disease. We focused our study on the purinergic P2X7 receptor (P2X7R), a sensor of ATP that is released from necrotic cells and that induces pro-inflammatory cytokine production and cell death. Our data provide new insights into the pathogenesis of severe tuberculosis by showing that mice that lack P2X7R have attenuated disease with substantially reduced bacillus dissemination and lung inflammation without evidence of necrosis.

## Introduction

Tuberculosis (TB) is a serious public health issue, with nearly 9 million new annual cases of the disease worldwide and 1.3 million deaths per year [1]. A third of the global population is infected with bacteria of the *Mycobacterium tuberculosis* complex (*Mycobacterium tuberculosis* – *Mtb* and *Mycobacterium bovis* – *Mbv*), although most effectively control the pathogen and remain asymptomatic by establishing equilibrium with the bacilli [2]. Mycobacteria are typically transmitted by aerosols and reach the lungs, where macrophages and other immune cells are recruited during the early innate response to infection. The latent infection results from the equilibrium between mycobacteria and the host defenses, in which inflammatory cells become organized as primary granulomas.

The reactivation of latent infections in immunocompetent individuals occurs at rates that range from 3% to 10% per lifespan [3]; these rates are dramatically increased by co-infection with the human immunodeficiency virus (HIV) [4]. Accelerations in active TB reactivations have also been described after the administration of tumor necrosis factor (TNF)α/IL-12/IL-23 blockers, which are used to treat inflammatory diseases, such as rheumatoid arthritis and Crohn's disease [2], [5]. Progressive primary TB is an alternative form of the disease that represents less than 10% of active TB cases in normal adults, although it is common in children less than 5 years of age and in immunocompromised individuals [6]. The aggressive forms of mycobacterial infections are characterized by the rapid expansion of primary granulomatous infiltrates in the lungs that results in tuberculous pneumonia and disseminated disease, such as miliary TB pneumonia. The precipitation of TB pathology is hallmarked by the formation of central caseous necrotic lesions filled with extracellular infectious bacilli and cell debris [7]. The factors that determine the transition of mycobacterial infections to active TB and the rapid progression of the disease have not been fully elucidated. The characteristics of the bacteria (i.e., high bacterial virulence or high dose of infection) and the host (i.e., genetic predisposition, immunodeficiency or malnutrition) are thought to contribute to progression of the disease [8].

Mouse TB models have been extremely useful in the characterization of mechanisms that are involved in disease pathogenesis and the generation of protective immunity. However, it is difficult to determine the factors that are responsible for the development of pulmonary necrosis because this process is atypical in mice that are infected with virulent mycobacteria, such as the H37Rv reference strain [7]. Necrotic lesions occur mostly in mice that are deficient in immunologically relevant molecules, such as inducible nitric oxide synthase (iNOS), the T cell receptor (TCR)αβ, interferon (IFN)γ, the TNF receptor (TNFR)-1, IL-6 and IL-12 [9]. Mice deficient in IL-1R1, IL-6, IL-10, CD4, CD8 or γ/δ T cells present with discrete pulmonary intragranulomatous necrosis. Alternatively, artificial conditions are required to induce necrosis in mice, such as intraperitoneal infections with extremely high numbers of bacilli [10] or intranasal infections with a low bacillus inoculum, followed by the administration of lipopolysaccharide (LPS) or polyinosinic-polycytidylic acid (Poly-IC) [9], [11], [12]. Moreover, dermal infections of iNOS-deficient mice lead to the formation of non-necrotizing granulomas in the lungs, which exhibit central caseation and hypoxia when IFNγ or TNFα are blocked [13].

Recent epidemiologic studies have shown that *Mtb* strains belonging to the emerging Beijing genotype family, which are associated with high virulence, transmissibility and drug resistance [14], are more likely to cause rapid progression of the primary infection to active TB than the strains belonging to other genotype groups [15]. Highly virulent Beijing strains, in contrast to the less virulent strains of Beijing or non-Beijing *Mtb* genotypes, promote unusual rapid fatal diseases in infected mice that are characterized by a severe pulmonary pathology, with high bacterial burden and extensive lung consolidation and areas of focal necrosis [16], [17], [18]. Furthermore, infections of mice with few *Mbv* strains are able to cause sudden pneumonia with extensive necrosis that lead to early death [19]. Studies with mice infected with hypervirulent mycobacterial strains suggest a possible method for investigating the specifics of the immunopathogenesis behind the aggressive forms of TB. An elucidation of the molecular mechanisms that are implicated in the development of severe TB is important to elaborate new therapeutic strategies.

Necrotic cell death permits the release of damage-associated molecular patterns (DAMPs), such as adenosine triphosphate (ATP). Extracellular ATP (eATP) is a danger signal for cells of the immune system and contributes to inflammatory and reparatory responses [20], [21], [22]. ATP is also released by activated and apoptotic cells through pannexin-1 hemichannels, which results in autocrine and paracrine cell signaling [23]. Some compelling evidence indicates that eATP levels increase *in vivo* to concentrations greater than 100 µM during pathological processes [24], [25]. Among the purinergic P2 receptors that recognize eATP, the P2X7 receptor (P2X7R) is uniquely able to induce pro-inflammatory cytokine production and cell death [26], [27], [28]. Therefore, eATP recognition by P2X7R contributes to the development of sterile and infectious inflammation in different experimental models [21], [22], [28], [29], [30]. The altered intracellular ionic milieu that results from P2X7R activation presumably triggers the nucleotide-binding domain and leucine-rich repeat-containing gene family pyrin domain-containing 3 protein (NLRP3) inflammasome, which leads to the pro-inflammatory cytokine production and apoptotic cell death [31], [32], [33]. The prolonged stimulation of P2X7R permits the formation of large transmembrane pores and the flux of molecules between 314 and 900 Da [26]. Depending on the magnitude and duration of the stimulus, the predominance of each of these molecular pathways dictates the development of apoptotic or necrotic cell death [34]. The combined activation of different molecular pathways culminates in pyroptosis, pyronecrosis or damaged mitochondria-dependent necroptosis [35].

Based on these findings, we were determined to investigate whether the recognition of eATP from necrotic cells by P2X7R could elicit an innate immune response and contribute to the development of severe TB. To investigate this possibility, we used murine models of pulmonary TB induced by intratracheal infections with the highly virulent isolates *Mtb* strain 1471 (Beijing genotype) and *Mbv* strain MP287/03. These mycobacterial strains were epidemiologically associated with outbreaks of TB in human and bovine populations, respectively, and showed high virulence in our previous *in vitro* studies [36], [37], [38]. In C57BL/6 mice that were infected with a low dose (approx. 100 bacilli) of hypervirulent mycobacteria, we reproduced several pathological manifestations of the aggressive forms of human TB, including tuberculous pneumonia with areas of focal necrosis and miliary dissemination to the liver and spleen. We then examined the courses of infection, lung pathologies and bacterial burdens in P2X7R^−/−^ and C57BL/6 mice. Our data provide new insights into the pathogenesis of severe TB by showing a requirement for P2X7R in the induction of macrophage death and the progression of tuberculous pneumonia, necrotic pulmonary lesions, bacillus dissemination and anticipated animal death.

## Results

### Severe TB caused by hypervirulent Beijing 1471 *Mtb* and MP287/03 *Mbv* is attenuated in P2X7R-deficient mice

Because ATP released from necrotic cells activates P2X7R and subsequently the innate immune response [23], [26], [27], we wondered if this molecular pathway may contribute to the development of the aggressive forms of TB that are associated with intense pulmonary necrosis. To investigate this possibility, two hypervirulent *Mtb* and *Mbv* strains were selected from 9 strains (*Mtb* – zt272, 1471 (genotype Beijing), 1777 (genotype LAM) and H37Rv; *Mbv* – AN5, B2, B29, MP287/03 and MP389/03) on the basis of the ability to induce severe TB, i.e., intense pulmonary infiltration with lung necrosis and precocious death of C57BL/6 mice that were infected intratracheally (i.t.) with low doses of bacilli (approx. 100 forms). Beijing 1471 *Mtb* and MP287/03 *Mbv* exhibited higher virulence within each subgroup (*Mtb* and *Mbv*) compared to the other strains (data not shown). The diseases induced by these strains in C57BL/6 and P2X7R^−/−^ mice were compared to those induced by the virulent H37Rv *Mtb* strain, which did not induce pulmonary necrosis in C57BL/6 mice. A previous study in P2X7R^−/−^ mice reported no involvement of the P2X7R during pulmonary H37Rv *Mtb* infection [39], whereas another study described a protective role for this receptor [40].

**Figure 1:**
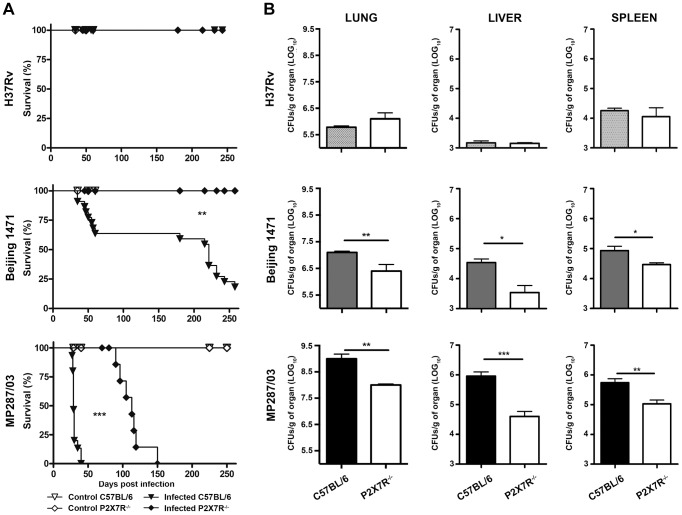
Survival curves and bacillary burdens in the lungs, liver and spleen of C57BL/6 and P2X7R^−/−^ mice infected with hypervirulent mycobacteria. C57BL/6 and P2X7R^−/−^ mice were infected i.t. with approx. 100 bacilli of H37Rv *Mtb*, Beijing 1471 *Mtb* and MP287/03 *Mbv*. Non-infected mice were used as controls. (A) The infected mice were examined daily to determine the survival curves (*n* = 13–19). (B) The number of CFU/g of tissue was evaluated in the left lung, liver and spleen on day 28 p.i. (means ± SD, *n* = 9). Significant differences were observed for the indicated groups (*p<0.05, **p<0.01 and ***p<0.001). The data are representative of three separate experiments.

Our results show that infection with Beijing 1471 *Mtb* killed 90% of C57BL/6 mice until 250 days post-infection (p.i.), whereas MP287/03 *Mbv* induced 100% mortality before day 40 p.i. (Figure 1A). Remarkably, all of the P2X7R^−/−^ mice infected with Beijing 1471 *Mtb* remained alive throughout this period, and the survival time was considerably increased when these animals were infected with MP287/03 *Mbv*. For the H37Rv *Mtb* infection, 100% survival was observed in C57BL/6 and P2X7R^−/−^ groups. The bacillus counts in the lungs of C57BL/6 mice at day 28 p.i. with Beijing 1471 *Mtb* and MP287/03 *Mbv* (CFU Log_10_ 6.7±0.2 and 8.8±0.2, respectively) were approximately 10-fold and 1,000-fold higher, respectively, than those in mice infected with H37Rv *Mtb* (CFU Log_10_ 5.8±0.4) (Figure 1B). Furthermore, the P2X7R^−/−^ mice infected with hypervirulent mycobacteria showed a nearly 10-fold reduction of lung bacillary burden compared to the C57BL/6 counterparts. In contrast, similar CFU values were obtained for the lungs of the C57BL/6 and P2X7R^−/−^ mice infected with H37Rv *Mtb*. Moreover, the disease dissemination to the liver and spleen was attenuated in P2X7R^−/−^ mice infected with hypervirulent mycobacteria, whereas no significant difference was found between C57BL/6 and P2X7R^−/−^ mice infected with H37Rv *Mtb*.

### P2X7R mediates pneumonia and lung necrosis in mice infected with hypervirulent mycobacteria

**Figure 2:**
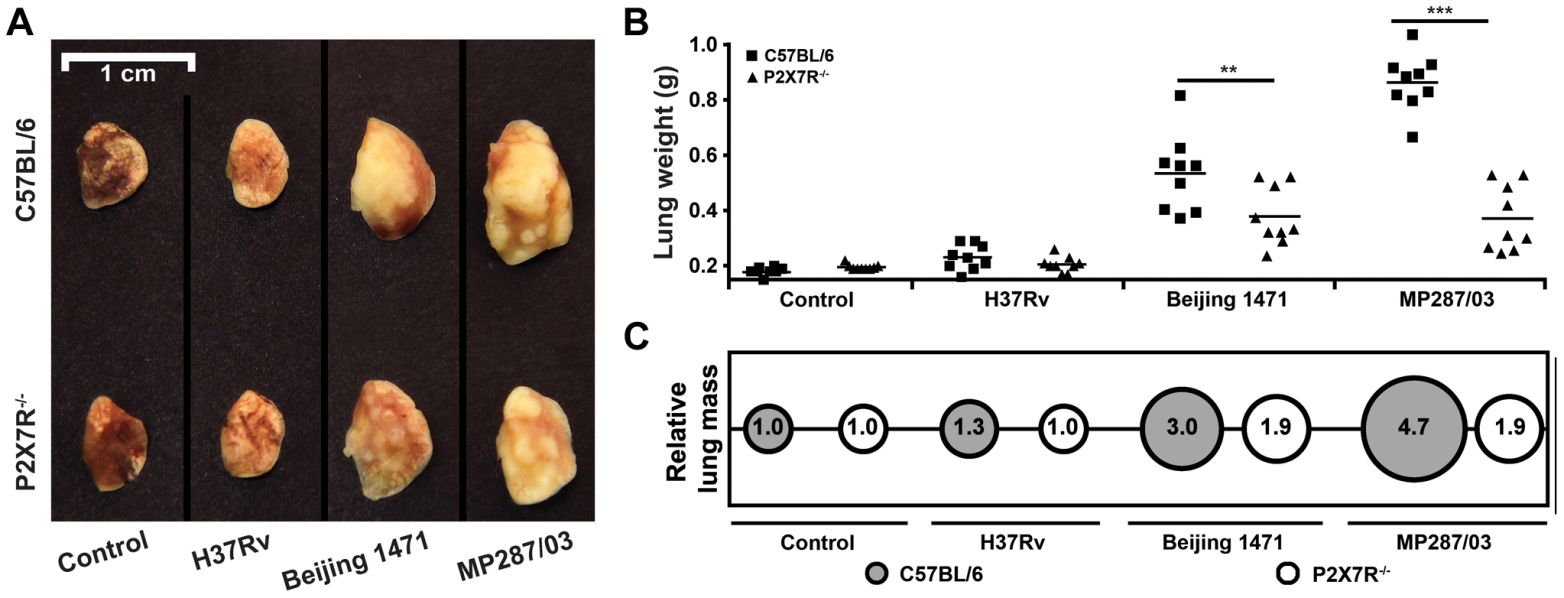
Lung gross pathology in C57BL/6 and P2X7R^−/−^ mice on day 28 p.i. with hypervirulent mycobacteria. C57BL/6 and P2X7R^−/−^ mice were infected i.t. with approx. 100 bacilli of H37Rv *Mtb*, Beijing 1471 *Mtb* and MP287/03 *Mbv*. Non-infected mice were used as controls. (A) Representative images of the right lungs are shown. (B) Right lung weights and (C) relative masses (circles) were evaluated (*n* = 9). The mean values are represented by horizontal lines. The relative lung masses were calculated by the ratios of the mean values of the lung weights in the indicated groups and the control group. Significant differences were observed for the indicated groups (**p<0.01 and ***p<0.001). The data are representative of three separate experiments.

To further characterize the effects of P2X7R during severe TB caused by hypervirulent mycobacteria, pneumonia and lung necrosis were evaluated in C57BL/6 and P2X7R^−/−^ mice on day 28 p.i. with the Beijing 1471 *Mtb*, MP287/03 *Mbv* and H37Rv *Mtb* strains. Macroscopically, the lungs had white nodules that were more prominent in C57BL/6 mice infected with hypervirulent mycobacteria (Figure 2A). Furthermore, the lung weights and relative masses were greatest in C57BL/6 mice infected with MP287/03 *Mbv*, followed by those infected with Beijing 1471 *Mtb* (Figures 2B and 2C). Considerably smaller white nodules were observed in the lungs of P2X7R^−/−^ mice that were infected with either hypervirulent strain, and these lungs were observed to have lower weights and relative masses. The lung weights in C57BL/6 and P2X7R^−/−^ mice infected with H37Rv *Mtb* did not statistically differ from those in non-infected controls.

**Figure 3:**
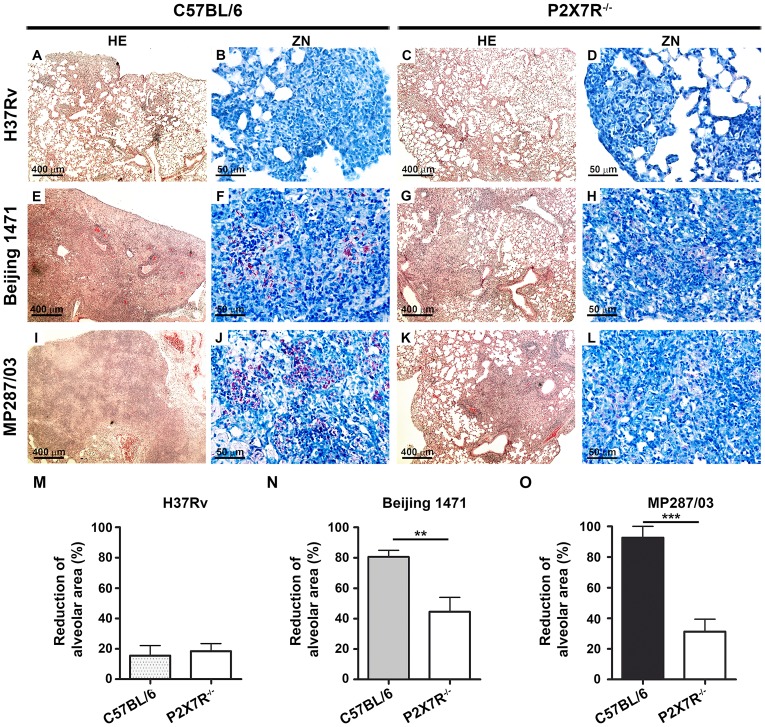
Lung histopathology in C57BL/6 and P2X7R^−/−^ mice on day 28 p.i. with hypervirulent mycobacteria. C57BL/6 and P2X7R^−/−^ mice were infected i.t. with approx. 100 bacilli of H37Rv *Mtb*, Beijing 1471 *Mtb* and MP287/03 *Mbv*. (A–L) Images show representative lung sections stained with HE (50× magnification; bar scales correspond to 400 µm) and ZN (400× magnification; bar scales correspond to 50 µm) methods. (A and C) H37Rv *Mtb*-infected C57BL/6 and P2X7R^−/−^ mice exhibited incipient granulomas. (B and D) Amplification of the inflamed areas shows no visible BAAR in the mice presented in B and D. (E and I) Beijing 1471 *Mtb*- and MP287/03 *Mbv*-infected C57BL/6 mice displayed extensive lung TB lesions with areas of necrosis. (F and J) Numerous BAARs were observed in the lesions described in E and I. (G and K) Beijing 1471 *Mtb*- and MP287/03 *Mbv*-infected P2X7R^−/−^ mice presented with small lung lesions with no visible necrosis. (H and L) Reduced amounts of BAARs were observed in the lesions described in G and K. (M) Morphometric quantification of lung sections shows a reduction in the intralveolar areas in infected mice compared to non-infected controls (means ± SD, *n* = 3–5). Significant differences were observed for the indicated groups (**p<0.01 and ***p<0.001). The data are representative of three separate experiments.

Microscopically, the lungs of C57BL/6 and P2X7R^−/−^ mice infected with H37Rv *Mtb* presented with incipient granulomas with no visible acid-alcohol resistant bacillus (BAAR) (Figures 3A–B and 3C–D, respectively). Additionally, comparable reductions of nearly 20% in the areas of lung sections covered by intralveolar spaces were observed in C57BL/6 and P2X7R^−/−^ mice infected with H37Rv *Mtb* (Figure 3M). In contrast, extensive cellular infiltrations in the lung tissues and intralveolar spaces, accompanied by granuloma formation with necrotic lesions and BAARs, were observed in C57BL/6 mice infected with Beijing 1471 *Mtb* (Figures 3E and 3F) and MP287/03 *Mbv* (Figures 3I and 3J). Remarkably, reduced areas of inflammation and necrosis with lower numbers of BAARs were observed in the lungs of P2X7R^−/−^ mice infected with both hypervirulent strains (Figures 3G–H and 3K–L). Accordingly, C57BL/6 mice infected with Beijing 1471 *Mtb* and MP287/03 *Mbv* showed sharp decreases of over 80% in the areas of lung sections occupied by intralveolar spaces, a phenomenon that was drastically attenuated in the P2X7R^−/−^ counterparts (Figure 3M).

### P2X7R mediates the recruitment of myeloid cells to the lungs in mice infected with hypervirulent mycobacteria

To determine the mechanisms by which the P2X7R detrimentally affects the outcome of severe TB, we next examined whether the lack of P2X7R modifies the pulmonary influx of leukocytes and the cytokine profile. We evaluated IL-1β production because it can be induced following P2X7R activation. We also examined IFNγ and IL-10 production because these cytokines have counterbalancing effects on inflammatory processes. These three cytokines have important roles in pulmonary TB [9], [13], [41].

**Figure 4:**
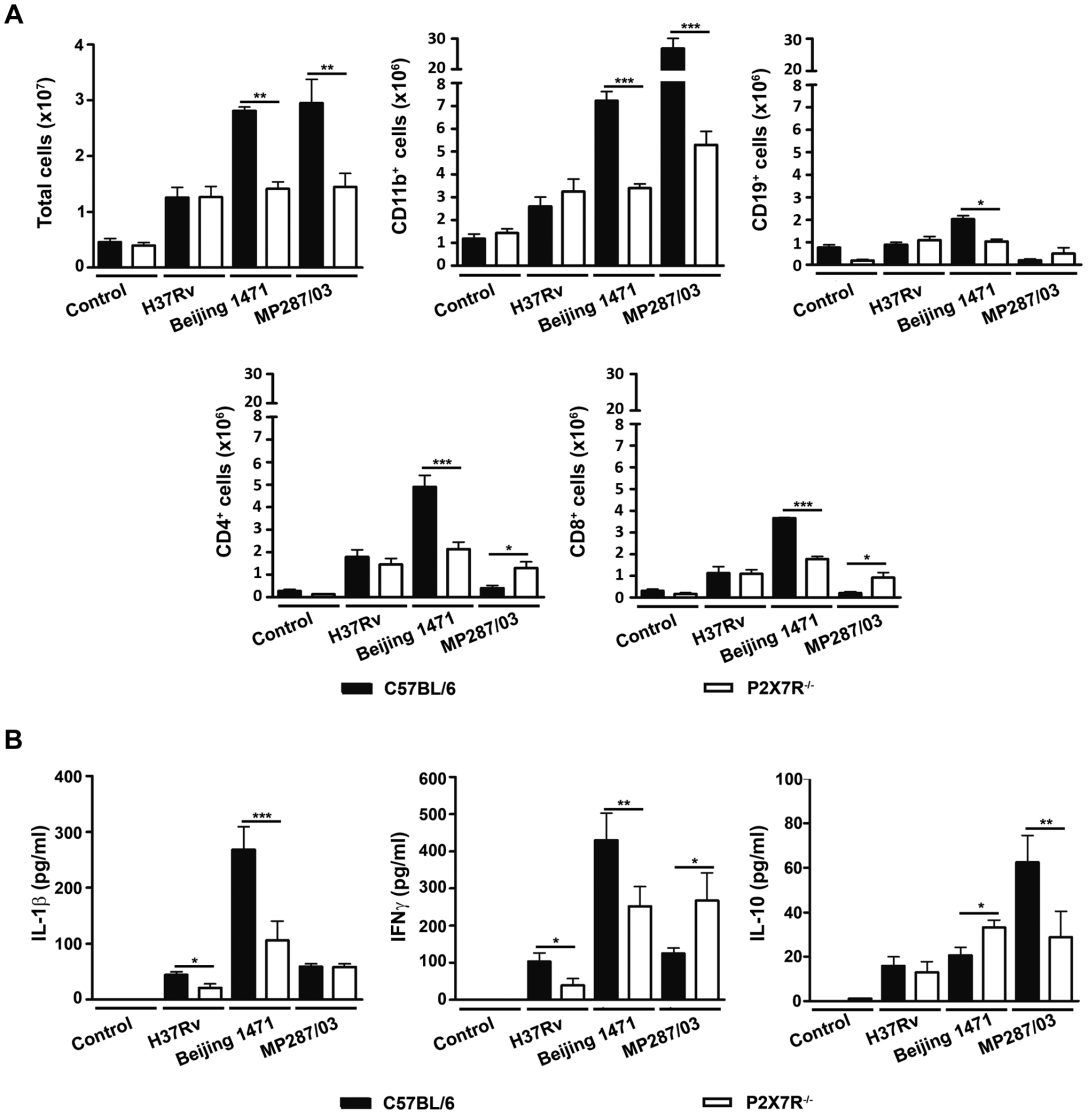
Lung infiltrating cells and cytokine production in C57BL/6 and P2X7R^−/−^ mice on day 28 p.i. with hypervirulent mycobacteria. C57BL/6 and P2X7R^−/−^ mice were infected i.t. with approx. 100 bacilli of H37Rv *Mtb*, Beijing 1471 *Mtb* and MP287/03 *Mbv*. Non-infected mice were used as controls. (A) The numbers of total, CD11b^+^, CD19^+^, CD4^+^ and CD8^+^ cells in the lungs are shown (means ± SD, *n* = 3). (B) IL-1β, IFNγ and IL-10 were quantified in the supernatants of lung cells after 48 h of culture (means ± SD, *n* = 3). Significant differences were observed for the indicated groups (*p<0.05, **p<0.01 and ***p<0.001). The data are representative of three separate experiments.

In agreement with the macro- and microscopic analysis of the lungs, C57BL/6 mice infected with Beijing 1471 *Mtb* and MP287/03 *Mbv* presented on day 28 p.i. with high numbers of lung infiltrating cells that were significantly reduced in P2X7R^−/−^ counterparts (Figure 4A). For the H37Rv *Mtb* infection, the total cell numbers in the lungs of C57BL/6 mice were similar to those of P2X7R^−/−^ mice and were 2- to 3-fold lower than those induced by hypervirulent strains. Unexpectedly, the composition of cellular infiltrates differed between the two groups of C57BL/6 mice infected with hypervirulent strains. Pulmonary myeloid (CD11b^+^ cells) and lymphoid (CD19^+^, CD4^+^ and CD8^+^ cells) subsets were found in similar proportions in Beijing 1471 *Mtb*-infected C57BL/6 mice. In contrast, a strong predominance of CD11b^+^ cells with virtually no T and B cells was observed in MP287/03 *Mbv*-infected C57BL/6 mice. The absence of P2X7R led to reduced pulmonary infiltration of myeloid cells in both hypervirulent infections and reduced lymphoid cells in the Beijing 1471 *Mtb* infection. However, MP287/03 *Mbv*-infected P2X7R^−/−^ mice presented with higher numbers of CD4^+^ and CD8^+^ cells compared to their C57BL/6 counterparts. C57BL/6 and P2X7R^−/−^ mice infected with H37Rv *Mtb* showed comparable numbers of pulmonary myeloid and lymphoid subsets. The CD11b^+^ population was mostly composed of Ly6C^+^ cells (monocytes) in all of the infected mice, with the exception of MP287/03 *Mbv*-infected C57BL/6 mice, which showed a predominance of Ly6G^+^ cells (neutrophils) (data not shown, manuscript in preparation).

The profiles of cytokines produced by lung infiltrating cells also varied between the two groups of C57BL/6 mice on day 28 p.i. with hypervirulent strains; relatively high or low production levels of pro-inflammatory cytokines were observed during severe TB (Figure 4B). More precisely, cells from Beijing 1471 *Mtb*-infected C57BL/6 mice produced higher levels of IL-1β and IFNγ. In contrast, cells from MP287/03 *Mbv*-infected mice secreted comparatively lower levels of pro-inflammatory cytokines and higher levels of IL-10, which supports the notion that MP287/03 *Mbv* is a poor inducer of pro-inflammatory cytokines [38]. As observed for the composition of cellular infiltrates, the absence of P2X7R had different effects on the cytokine profiles induced by hypervirulent strains. For the Beijing 1471 *Mtb* infection, cells from P2X7R^−/−^ mice secreted significantly reduced amounts of IL-1β and IFNγ and increased amounts of IL-10 compared to cells from C57BL/6 mice. For the MP287/03 *Mbv* infection, comparable low levels of IL-1β were found in cell supernatants from C57BL/6 and P2X7R^−/−^ mice, but the lack of P2X7R resulted in an increase of IFNγ and a decrease of IL-10. The H37Rv *Mtb* infection induced very low production of IL-1β, IFNγ and IL-10; the amount of pro-inflammatory cytokines was significantly reduced in the absence P2X7R.

### Hypervirulent mycobacteria induce macrophage necrosis by a P2X7R-mediated mechanism

The following experiments were performed to further elucidate the mechanisms that are responsible for the deleterious role of P2X7R in severe TB. The intracellular bacterial growth and dissemination were investigated in bone marrow-derived macrophages (BMDMs) obtained from C57BL/6 and P2X7R^−/−^ mice that were infected with H37Rv *Mtb*, Beijing 1471 *Mtb* and MP287/03 *Mbv* strains. The analysis was performed over a maximum period of 6 days in culture after macrophage differentiation, during which the viability of non-infected BMDMs was maintained above 95%. The intracellular bacterial growth was evaluated at an MOI (multiplicity of infection) of 1, which ensured bacillus multiplication for 6 days p.i. in the absence of spontaneous and bacillus-induced macrophage death. The low MOI was particularly important for hypervirulent mycobacterial strains that grew rapidly and killed the macrophages. The macrophage necrosis induced by the mycobacteria was systematically determined at MOIs of 10 and 20; the best conditions to discriminate C57BL/6 and P2X7R^−/−^ BMDMs are shown.

**Figure 5:**
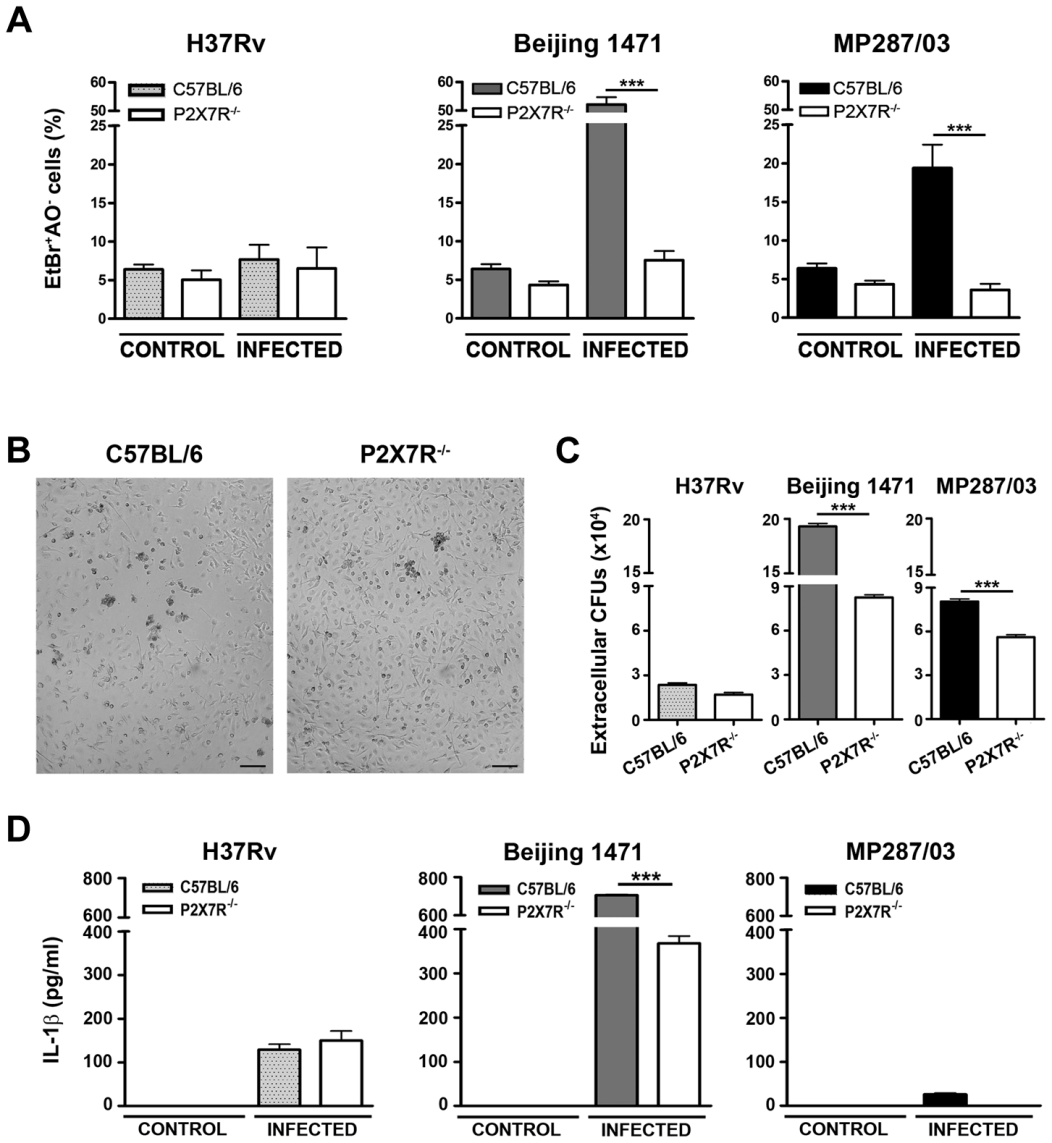
Induction of necrotic cell death and the release of bacteria and IL-1β in P2X7R^−/−^ and C57BL/6 BMDMs infected with hypervirulent mycobacteria. C57BL/6 and P2X7R^−/−^ BMDMs were infected with H37Rv *Mtb*, Beijing 1471 *Mtb* and MP287/03 *Mbv* at an MOI of 10. (A) On day 4 p.i., necrotic cells were identified by AO/EthBr incorporation. (B) Images show C57BL/6 and P2X7R^−/−^ BMDMs on day 4 p.i. with Beijing 1471 *Mtb* (200× magnification; bar scales correspond to 100 µm). (C) The release of mycobacteria was examined by CFU quantification in the supernatants of the infected cultures described in A. (D) On day 3 p.i., IL-1β was quantified in the culture supernatants. Significant differences were observed for the indicated experimental conditions (***p<0.001). The data represent the means ± SD of samples in triplicate. The data are representative of three separate experiments.

The hypervirulent mycobacterial strains multiplied inside C57BL/6 BMDMs much faster than did H37Rv *Mtb* and were the most effective strains for inducing cell death (Figure S1A and S1B). In an experimental condition of low bacillus-induced macrophage death, P2X7R did not interfere with intracellular bacterial growth; the growth kinetics of the mycobacterial strains within C57BL/6 and P2X7R^−/−^ BMDMs were similar at an MOI of 1 (Figure S1A). Remarkably, negligible numbers of necrotic P2X7R^−/−^ BMDMs were observed on day 4 p.i. with hypervirulent mycobacteria at an MOI of 10, an experimental condition that induced C57BL/6 BMDM death (Figures 5A and 5B). The kinetics of cellular viability at an MOI of 20 showed that the lack of P2X7R protected the BMDMs from early death, but the Beijing 1471 *Mtb* infection killed C57BL/6 and P2X7R^−/−^ BMDMs on day 6 p.i. (Figure S1B). Our data also show that P2X7R-mediated necrosis of macrophages facilitates the release of hypervirulent mycobacteria; more viable Beijing 1471 *Mtb* and MP287/03 *Mbv* bacilli were detected in the supernatants of C57BL/6 BMDMs compared to those of P2X7R^−/−^ BMDMs (Figure 5C). Supporting the idea that Beijing 1471 *Mtb* and MP287/03 *Mbv* infections induce macrophage necrosis, the majority of dead cells on day 4 p.i. with hypervirulent strains were stained with PI but not with annexin V (Figure S2). Furthermore, in accordance with the *in vivo* data (Figure 4B), P2X7R^−/−^ BMDMs that were infected with Beijing 1471 *Mtb* produced lower levels of IL-1β than did the counterpart C57BL/6 BMDMs (Figure 5D). MP287/03 *Mbv* was a poor inducer of IL-1β production in C57BL/6 BMDMs, and this cytokine was not detected in the supernatants from P2X7R^−/−^ BMDMs. The infection of C57BL/6 and P2X7R^−/−^ BMDMs with H37Rv *Mtb* caused negligible macrophage death (Figure 5A), extracellular release of the bacilli (Figure 5C) and IL-1β production (Figure 5D), which were not influenced by the P2X7R.

### For hypervirulent strains but not for H37Rv *Mtb*, eATP stimulation causes a P2X7R-mediated release of viable mycobacteria by infected macrophages

Previous studies show that engagement of the P2X7R promotes phagosome-lysosome fusion and induces apoptosis, thereby causing intracellular mycobacterial killing [42], [43], [44]. In agreement with the protective role of the P2X7R against mycobacterial infection, macrophages from subjects homozygous for loss-of-function P2X7R polymorphisms exhibit ablated eATP-induced apoptosis and killing of mycobacteria [45], [46], [47]. As these analyses were performed with BCG, an avirulent *Mbv* strain, the apparent contrast between the protective role of P2X7R reported in these studies and the deleterious role of P2X7R described here may reside in the varying virulence of mycobacteria. If this is the case, eATP stimulation of the P2X7R may have different consequences in macrophages infected with virulent or hypervirulent strains, causing mycobacterial killing or dissemination, respectively. To examine this possibility, the numbers of intracellular and extracellular bacilli were determined in P2X7R^−/−^ and C57BL/6 BMDMs that were infected with H37Rv *Mtb*, Beijing 1471 *Mtb* and MP287/03 *Mbv* strains and then stimulated with eATP. The protocol used in these experiments was similar to those employed in the previous *in vitro* analyses described above [45], [46], [47].

**Figure 6:**
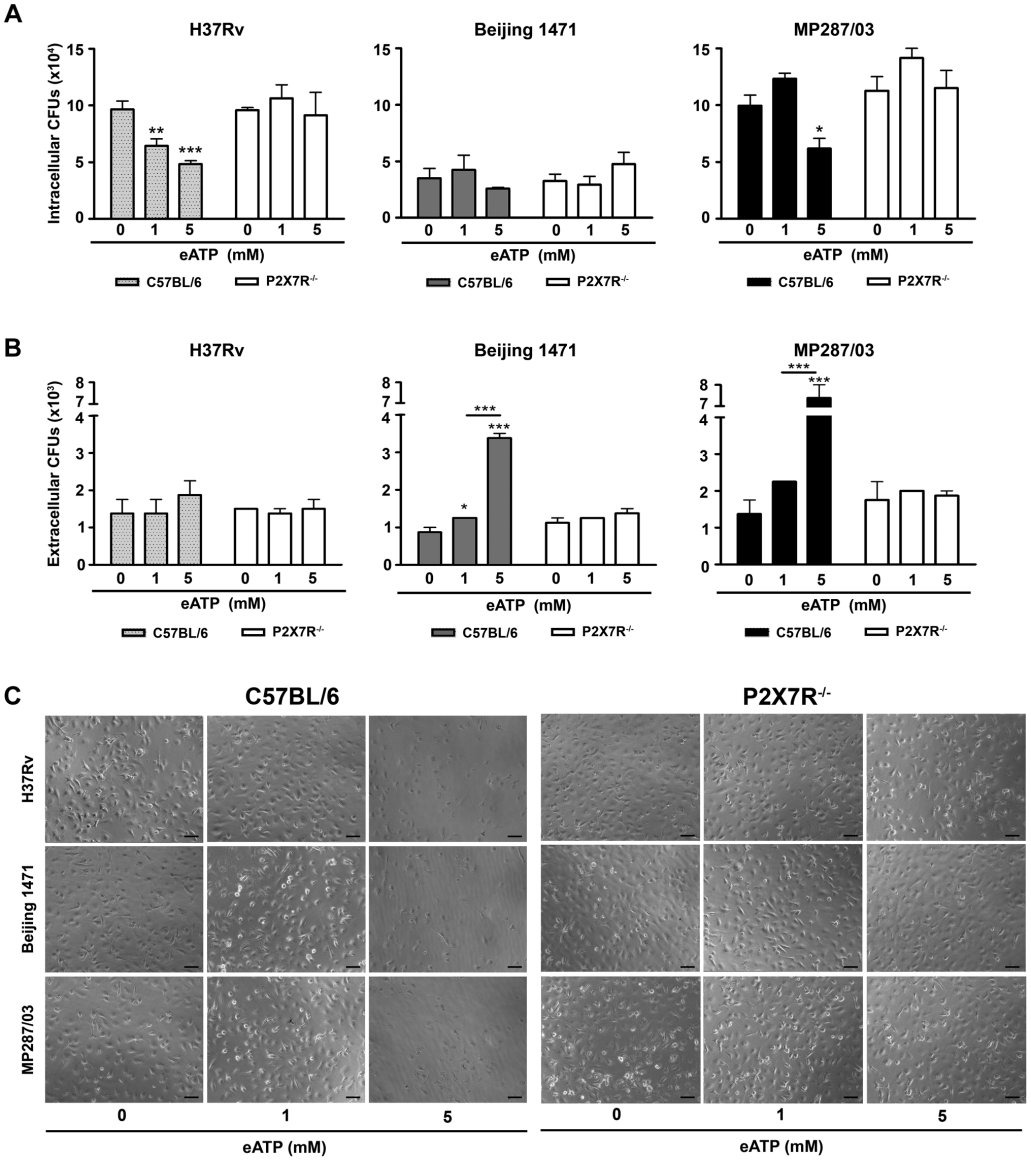
Effects of eATP on mycobacterial killing or release by C57BL/6 and P2X7R^−/−^ BMDMs. C57BL/6 and P2X7R^−/−^ BMDMs were infected with H37Rv *Mtb*, Beijing 1471 *Mtb* and MP287/03 *Mbv* at an MOI of 10; treated with 0, 1 or 5 mM eATP for 30 min; and analyzed 6 h later. (A) Mycobacterial killing was evaluated by intracellular CFU quantitation. (B) The release of mycobacteria from necrotic cells was examined by CFU quantification in the culture supernatants. (C) Images show BMDM cultures (200× magnification; bar scales correspond to 100 µm). Significant differences were observed between eATP-treated and non-treated groups and for the indicated experimental conditions (*p<0.05, **p<0.01 and ***p<0.001). The data represent the means ± SD of samples in triplicate. The data are representative of three separate experiments.

The activation of H37Rv *Mtb*-infected C57BL/6 BMDMs with 1 and 5 mM eATP led to P2X7R-mediated decreases in the numbers of intracellular bacilli and no increase in the numbers of extracellular bacilli (Figures 6A and 6B). At 1 mM eATP, macrophage activation rather than apoptosis may determine the P2X7R-mediated killing of H37Rv *Mtb*. At this eATP concentration, C57BL/6 BMDMs exhibited a typical morphology of viable macrophages (Figure 6C). In contrast, at 5 mM eATP, the protective effect was associated with a high level of death of H37Rv *Mtb*-infected C57BL/6 BMDMs. Indeed, most C57BL/6 BMDMs presented with a necrotic phenotype at concentrations greater than 3 mM eATP (Figure S3). As expected, P2X7R^−/−^ BMDMs were resistant to eATP-induced death. For hypervirulent strains, the effects of eATP stimulation on C57BL/6 BMDMs were completely different than they were for the H37Rv *Mtb* strain. At 1 mM eATP, the numbers of intracellular and extracellular bacilli showed minor or no changes for both the Beijing 1471 *Mtb* and MP287/03 *Mbv* strains (Figures 6A and 6B). At 5 mM eATP, the P2X7R-mediated decrease in the amount of intracellular bacilli was associated with macrophage death (Figure 6C). Most importantly, significantly higher numbers of viable mycobacteria were detected in the supernatants of C57BL/6 BMDMs infected with the Beijing 1471 *Mtb* and MP287/03 *Mbv* strains (Figure 6B). The release of viable mycobacteria was mediated by the P2X7R, which indicated that macrophages had undergone P2X7R-mediated necrotic death. These results corroborate our supposition that eATP stimulation of infected macrophages has opposite consequences for virulent and hypervirulent strains. In other words, the heterogeneity of mycobacterial virulence explains the dichotomy concerning the protective and deleterious roles of P2X7R in pulmonary TB.

## Discussion

The development of two experimental mouse models of human-like severe TB allowed us to determine the general properties of phylogenetically distant strains of hypervirulent mycobacteria that are associated with poor prognosis. The ability of Beijing 1471 *Mtb* and MP287/03 *Mbv* to rapidly grow in the lungs, to cause pulmonary necrosis and to disseminate to other organs likely contributed to the worsening of the disease in C57BL/6 mice. These data emphasize the importance of the mycobacterial genetic background in the pathogenesis of severe TB and corroborate previous studies in mice that showed significant variability in the bacillus virulence of *Mtb* strains that were isolated from TB patients [19], [18], [48], [49] or *Mbv* strains that were isolated from infected cattle [19], [48], [49]. Additionally, the comparison of the disease progression in C57BL/6 and P2X7R^−/−^ mice that were infected i.t. with a low inoculum (approx. 100 bacilli) of H37Rv *Mtb*, Beijing 1471 *Mtb* and MP287/03 *Mbv* allowed us to describe the crucial role of P2X7R in the aggressive forms of pulmonary TB. These findings provide a new insight into the pathogenesis of severe TB by showing that mice that lack P2X7R have attenuated disease with substantially reduced bacillus burdens in the lungs, livers and spleens. The absence of P2X7R also promoted significant reductions in pneumonia and lung necrosis on day 28 p.i. with Beijing 1471 *Mtb* and MP287/03 *Mbv*, which corroborates our hypothesis of DAMP involvement in the pathogenesis of severe TB. Furthermore, our data are consistent with a previous study in P2X7R^−/−^ mice that reported no role for P2X7R during pulmonary H37Rv *Mtb* infection [39].

P2X7R signaling could detrimentally affect the outcome of severe TB by inducing the production of IL-1β and exacerbating the pulmonary influx of pathogen-permissive monocytes and macrophages. A recent study has implicated the recruitment of CD11b^+^F4/80^+^Gr1^int^ myeloid cells to the lungs in the exacerbation of H37Rv *Mtb* infection in mice treated with Poly-IC [12]. Accordingly, our data link the reduction in the pulmonary infiltration of CD11b^+^ myeloid cells with disease attenuation in P2X7R^−/−^ mice that were infected with Beijing 1471 *Mtb* and MP287/03 *Mbv* strains. The lack of P2X7R during the Beijing 1471 *Mtb* infection also resulted in lower IL-1β production by lung infiltrating cells, which could have been responsible for the secretion of less IFNγ and more IL-10. Previous studies showing the resistance of hypervirulent mycobacteria to IFNγ-mediated intracellular killing [37], [38] may explain the ineffectiveness of IL-1β, a critical cytokine in the protection against H37Rv *Mtb* [41], in preventing the fatal outcome of the disease in Beijing 1471 *Mtb*-infected C57BL/6 mice. Nevertheless, the induction of IL-1β does not fully explain how P2X7R contributes to exacerbate the disease progression during MP287/03 *Mbv* infection. In this case, severe TB occurred concomitantly with a relatively high secretion of IL-10 and low secretion of IL-1 β and IFNγ, which correlated with low numbers of CD4^+^ and CD8^+^ T cells in the lungs. In fact, we have recently shown that MP287/03 *Mbv*-infected C57BL/6 macrophages are biased towards an M2 immune response profile and induce higher arginase-1 expression and reduced NO production compared to H37Rv *Mtb*-infected macrophages [38]. The inhibition of innate immune responses was also described for other mycobacterial strains [18], [50], [51], and it has been considered to be a selective advantage of modern mycobacterial strains that contributes to rapid disease progression and transmission [51]. Furthermore, the lack of the P2X7R during MP287/03 *Mbv* infection did not affect the low production of IL-1β by lung infiltrating cells, but it restored the CD4^+^ and CD8^+^ T cell responses with the secretion of more IFNγ and less IL-10.

An alternative insight into the mechanism responsible for the prejudicial role of P2X7R during severe TB was provided by the *in vitro* analysis of C57BL/6 and P2X7R^−/−^ macrophages that were infected with the H37Rv *Mtb*, Beijing 1471 *Mtb* and MP287/03 *Mbv* strains. The abilities of hypervirulent mycobacteria to rapidly grow in C57BL/6 mice and to cause pulmonary necrosis were associated with the abilities to multiply vigorously within C57BL/6 macrophages *in vitro* and to induce cell death. These findings show a parallel between the *in vivo* and *in vitro* behavior of the mycobacterial strains as reported in other studies [37], [52]. In a recapitulation of the *in vivo* data, only Beijing 1471 *Mtb* elicited the production of high levels of IL-1β in C57BL/6 macrophages, but both hypervirulent mycobacteria strains induced cell death. The subsequent release of live bacilli and the negative staining for annexin V clearly establish that these infected macrophages undergo necrotic cell death. Therefore, with consideration for the critical role of the NLRP3 inflammasome in *Mtb*-induced necrotic death [53], it is likely that signaling through P2X7R accelerates this process in C57BL/6 macrophages that are infected with Beijing 1471 *Mtb* and MP287/03 *Mbv*. Accordingly, the absence of the P2X7R led to a delay in macrophage death that was accompanied by lower release of hypervirulent mycobacteria. This finding indicates that P2X7R cooperates with mycobacterial components exhibiting membrane-lysing activity, such as the ESAT-6 protein from *Mtb* [54], a phenomenon that may occur through autocrine or paracrine ATP release. The membrane permeabilization that results as a secondary effect of P2X7R-mediated necrotic death might also have facilitated the release of IL-1β during the Beijing 1471 *Mtb* infection in C57BL/6 macrophages [55], as this response was partially inhibited in P2X7R^−/−^ macrophages. Thus, even if P2X7R-mediated macrophage death is an evolutionarily selected mechanism of host defense, in Beijing 1471 *Mtb* and MP287/03 *Mbv* infections, it allows the bacilli to escape from the innate host defenses and to spread to new cells. A similar behavior was recently described for mycobacteria that possess RD-1 virulence factors, which permit phagolysosomal rupture and allow access to the cytosol [56].

The deleterious role of P2X7R during severe TB implies that hypervirulent mycobacteria are resistant to the protective mechanisms that are elicited in macrophages following eATP stimulation. In avirulent BCG infection, the engagement of the P2X7R promotes phagosome-lysosome fusion and induces macrophage apoptosis, thereby causing mycobacterial death [42], [43], [44]. Accordingly, macrophages from subjects homozygous for loss-of-function P2X7R polymorphisms exhibit ablated eATP-induced apoptosis and eATP-mediated killing of BCG bacilli [45], [46]. In fact, our data clearly establish that eATP stimulation has opposite outcomes in macrophages that are infected with virulent or hypervirulent mycobacteria. Thus, eATP induced the P2X7R-mediated killing of H37Rv *Mtb* bacilli and the P2X7R-mediated release of viable Beijing 1471 *Mtb* and MP287/03 *Mbv* bacilli. The resistance of these hypervirulent mycobacteria to intracellular killing has also been described following macrophage stimulation with IFNγ [37], [38] and can be attributed to the ability of pathogenic mycobacteria to block phagosome-lysosome fusion [57]. The moderate extent of tissue damage with consequently small amounts of eATP release and the susceptibility to eATP-induced intracellular killing explain, respectively, the lack of involvement or the protective role of P2X7R during the H37Rv *Mtb* infection [39], [40]. Finally, several epidemiological studies reported that loss-of-function polymorphisms in the human P2X7R gene increase the risk of pulmonary TB [58], [59], [60], [61], [62]. However, opinions on this issue have not reached a consensus, and other studies show a lack of association between these parameters [63], [64]. In sum, we can envisage that the heterogeneity of the samples analyzed in these studies may account for the failure to reach a consistent conclusion. Namely, the risk of pulmonary TB in individuals with loss-of-function polymorphisms in the P2X7R gene may be increased for mild TB and decreased for severe TB. The protective role of the P2X7R functional deficiency in the aggressive forms of TB could explain why evolutionary pressure has maintained these gene polymorphisms at high rates in the human population.

**Figure 7:**
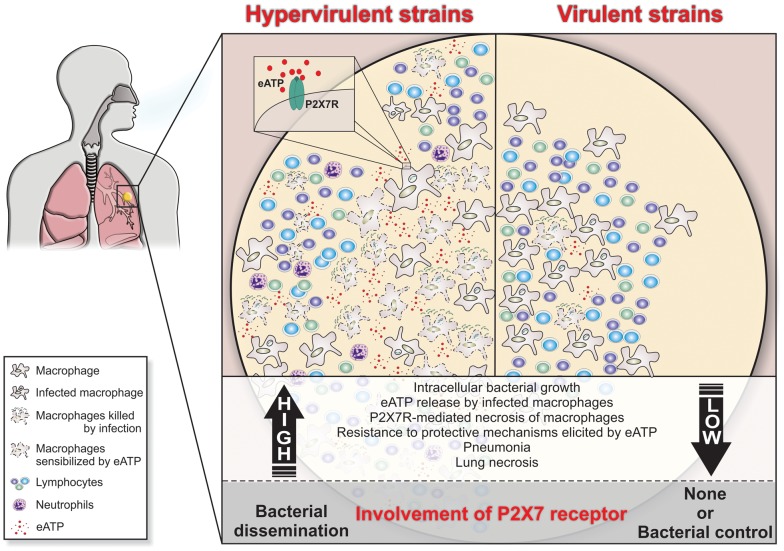
Schematic illustration of a hypothetical model to explain the high and low involvement of the P2X7R during severe and mild TB. In severe TB, the rapid intracellular growth of hypervirulent mycobacteria results in massive macrophage damage. The eATP released by damaged cells may engage the P2X7R on their surfaces or on neighboring cells. The autocrine or paracrine P2X7R signaling cooperates with mycobacterial components exhibiting membrane-lysing activity and accelerates the necrotic death of infected macrophages and the spread of bacilli. The resistance of hypervirulent mycobacteria to the protective mechanisms elicited in macrophages by eATP contributes to disease dissemination. The release of large amounts of eATP triggers a vicious cycle that exacerbates the pulmonary recruitment of pathogen-permissive monocytes and macrophages and thereby leads to further intracellular bacillus growth. The suppressive environment that results from an excess of adenosine, a byproduct of eATP degradation, may also facilitate the survival of hypervirulent mycobacteria. According to this model, lung necrosis derives from programmed cell death that is triggered by P2X7R signaling. The modest levels of tissue damage induced by less virulent strains and the susceptibility of these mycobacteria to eATP-induced intracellular killing explain, respectively, the minor role and the protective effect of the P2X7R in the mild forms of TB.

The data from two mouse models indicated P2X7R as a key molecule in the development of severe TB and suggested that lung necrosis is enhanced by the activation of this receptor. P2X7R appears to have a dual role in the development of aggressive TB. First, it might facilitate the dissemination of hypervirulent mycobacteria by inducing the lysis of infected macrophages. Second, it might contribute to pneumonia and lung necrosis by promoting widespread cell destruction. Based on these results, we propose that the massive destruction of macrophages by hypervirulent mycobacteria with the help of the P2X7R leads to the release of large amounts of ATP (Figure 7). This release triggers a vicious cycle in which eATP exacerbates the pulmonary influx of pathogen-permissive monocytes and macrophages [12] and leads to further cell destruction by a P2X7R-mediated mechanism. This scenario is made worse by the resistance of hypervirulent mycobacteria to the protective mechanisms elicited in macrophages by eATP. The suppressive environment that results from an excess of adenosine, a byproduct of eATP degradation, may also facilitate the survival of hypervirulent mycobacteria [23]. This study provides a perspective for the development of new therapeutic approaches in which drugs designed to inhibit the P2X7R are used to ameliorate the outcomes of aggressive forms of TB.

## Materials and Methods

### Mice

Male 8- to 10-week-old C57BL/6 and P2X7R^−/−^ (C57BL/6 background) mice (originally from the Jackson Laboratory, USA) were bred under specific pathogen-free conditions at the Isogenic Mice Facility (ICB/USP, Brazil). The P2X7R^−/−^ mice were generated by Pfizer Inc. (USA) and backcrossed to C57BL/6 mice for 7 generations; a single nucleotide polymorphism panel analysis throughout the genome suggested a C57BL/6 genetic background (http://jaxmice.jax.org/strain/005576.html). Once infected, the experimental groups were maintained in microisolator cages at the Biosafety Level 3 Mice Facilities (UENF and FCT-USP, Brazil) under controlled temperature and humidity and were fed *ad libitum*.

### Ethics statement

All experimental procedures were conducted in accordance with the national regulations on ethical guidelines for mouse experimentation and welfare of the Conselho Nacional de Saúde and Colégio Brasileiro de Experimentação Animal (COBEA, Brazil). The protocols were approved by the Animal Care Committee of ICB-USP and UENF under the permit numbers 0026/2009 and 0020/2006, respectively.

### Mycobacteria

The *Mtb* strain of the Beijing genotype (Beijing 1471) was isolated from a TB patient at the St. Petersburg Physiopulmonology Research Institute, Russia [37]. Dr. José Soares Ferreira Neto (Instituto de Medicina Veterinária, USP) and Dr. Philip Suffys (Fundação Oswaldo Cruz, Brazil) kindly provided the bovine *Mbv* isolate (MP287/03 - SB0295 spoligotyping) and the *Mtb* reference strain (H37Rv - ATCC), respectively. Mycobacteria from a single colony-forming unit (CFU) were suspended in Middlebrook 7H9 medium (Difco, BD Biosciences, USA) that was supplemented with 10% albumin-dextrose-catalase (ADC) (Difco) and 0.05% Tween 80 (Sigma-Aldrich, USA), with (for *Mbv*) or without (for *Mtb*) 0.4% sodium pyruvate (Sigma-Aldrich), and were frozen at −80°C in aliquots of 10^8^ bacilli/ml. Prior to performing the experiments, the aliquots were thawed, diluted in complete 7H9 medium, sonicated and incubated at 37°C for 7 days. To avoid bacterial clumps, the aliquots were sonicated for 1 min, homogenized and kept at rest for 10 min at room temperature (RT). The suspensions were collected, and the absence of bacterial clumps was monitored by microscopic examination. The bacilli were quantified by spectrophotometry at 600 nm.

### CFU counting

The live bacterial burdens were determined by serial dilutions and the plating of cell or tissue homogenates in Middlebrook 7H10 medium (Difco), which was supplemented with 10% oleic acid/albumin/dextrose/catalase (OADC) (Difco); 0.4% sodium pyruvate was added for the *Mbv* strains. CFU numbers were determined after a 3 week incubation at 37°C.

### Mouse infections

Mice were anesthetized by intraperitoneal injections of ketamine (Vetbrands, Brazil; 100 mg/kg) and xylazine (Vetbrands; 15 mg/kg), which were diluted in sterile saline. For each mouse, the trachea was exposed via a small midline incision, and 60 µl of the bacterial suspension (approx. 100 bacilli) was inoculated intratracheally (i.t.) [65]. The incision was sutured with sterile silk. The inoculum dose was confirmed by a CFU count of lung homogenates at 24 h p.i. and varied ±20% in relation to the dose of infection.

### Macroscopic and microscopic analyses of the lungs, liver and spleen

The relative mass (organ weight in infected mice/control mice) of each organ was determined. Tissue samples from the right lung, liver and spleen were fixed with 10% buffered formalin, photographed and embedded in paraffin. Serial 4–5-µm sections were stained with hematoxylin-eosin (HE) dye to analyze the tissue alterations and by the Ziehl-Neelsen (ZN) method to detect BAARs. The samples were examined with a Leica microscope (Germany), and images were captured with a Coolpix P995 Nikon camera (Japan).

### Morphometric analysis of lung tissues

The percentages of the lung tissue occupied by intralveolar space were determined as previously reported for intravascular space in the placenta [66]. Briefly, 8 randomly selected images were analyzed in each HE-stained section of lung tissue (50× magnification). Using ImageJ software (National Institutes of Health, USA), the images were transformed in gray scale (8-bit) and the areas corresponding to intralveolar space were colored in red. The reduction in the percentages of intralveolar area was calculated by the equation (x–y)*100/x, where x and y are, respectively, the percentages of pixels covered by red areas in the images of non-infected and infected mice.

### Isolation of lung infiltrating cells

The left lungs were washed with sterile PBS and placed in Petri dishes with RPMI 1640 medium (Gibco, USA). The dissected lung tissue was incubated in medium that contained Liberase (Sigma-Aldrich; 2 µg/ml) and type IV bovine pancreatic DNAse (Roche Diagnostic; 1 µg/ml) at 37°C for 45 min. Cells were dispersed with a 10-ml syringe (BD Biosciences) that was fitted with an 18-gauge needle and filtered with a cell strainer (Corning, USA). Red blood cells were depleted with a lysis buffer (0.144 M NH_4_Cl, 0.0169 M TRIS base, pH 7.4) at 37°C in a 5% CO_2_ atmosphere for 5 min.

### Phenotypic analysis of lung infiltrating cells

Lung infiltrating cells (1×10^6^) were stained using appropriate combinations of Pacific Blue-, APC-Cy7-, PE-Cy7-, PE- or V500-labeled monoclonal antibodies (mAbs) to CD11b (ICRF44), CD19 (1D3), CD4 (RM4-5) and CD8 (53-6.7) (BD Pharmingen, USA). Cells were fixed with 2% paraformaldehyde and analyzed by flow cytometry (FACSCanto, BD Biosciences) with FlowJo software.

### Cytokine quantification

The lung infiltrating cells (5×10^4^ cells/well) were suspended in complete RPMI 1640 medium (Gibco) with 1 mM sodium pyruvate, 2 mM glutamine, 0.05% gentamicin and 10% fetal calf serum (FCS) and were cultured in 96 well-plates (Corning) at 37°C in a 5% CO_2_ atmosphere for 48 h. Cell culture supernatants were harvested, filter sterilized and stored at −80°C. The levels of IL-1β, IFNγ and IL-10 in the cell culture supernatants were measured with a Fluorokine kit (R&D System, USA) and a Bioplex system (Bio-Rad, USA).

### BMDM generation

BMDMs were generated from mouse femurs as described previously [43], with some modifications. The two femurs were each flushed with 5 ml of complete DMEM-F12 medium (Gibco; 1 mM sodium pyruvate, 2 mM glutamine, 0.05% gentamicin and 10% FCS) and were incubated at 37°C in a 5% CO_2_ atmosphere for 12 h. Non-adherent cells were harvested and cultured for an additional 3 days in a cell culture flask (Corning; 75 cm^2^) that contained 10 ml of complete DMEM-F12 medium that was supplemented with 20% L929 cell-conditioned medium as a source of macrophage colony-stimulating factor. After 3 days, an additional 10 ml of L929 cell-conditioned medium was added. Four days later, BMDMs were detached from the flasks with 4 ml of 2 mM trypsin in PBS. The viability of BMDMs was higher than 95% over a week period and decreased thereafter.

### BMDM infection and eATP stimulation

The *in vitro* experiments were performed in a time window of 6 days after macrophage differentiation. BMDMs (4×10^4^ cells) were cultured in 96-well plates in DMEM-F12 medium with 2% FCS at 37°C in a 5% CO_2_ atmosphere. To evaluate intracellular bacterial growth, BMDMs were infected at an MOI of 1 for 3 h. After washing 3 times with PBS, the intracellular bacteria were evaluated by performing CFU counts of BMDMs that were lysed with 0.1% saponin (Sigma-Aldrich) for 10 min. To analyze the cytotoxicity induced by the bacteria, BMDMs were infected and cellular viability was determined by acridine orange and ethidium bromide (AO/EthBr; Sigma-Aldrich) incorporation using an inverted NIKON TS-100 fluorescence microscope (Japan). To establish the best experimental condition with which to discriminate the differences between experimental groups, the cellular viabilities were systematically determined at MOIs of 10 and 20. The effects of eATP on bacterial intracellular killing and release were assessed as previously described [45], [46], [47]. Briefly, BMDMs were infected at an MOI of 10, washed 3 times with PBS and stimulated with 1 or 5 mM eATP (Sigma-Aldrich) for 30 min. The intracellular and extracellular CFU counts were determined after 6 h in culture. The cytotoxicity induced by eATP in non-infected BMDMs was determined after 24 h in culture with 1 or 5 mM eATP. The cells were stained with AO/EthBr and analyzed in an inverted NIKON TS-100 fluorescence microscope.

### Annexin V staining

BMDMs (4×10^4^ cells) were infected at an MOI of 10, washed 3 times with PBS and cultured for 4 days in 96-well plates in DMEM-F12 medium with 2% FCS at 37°C in a 5% CO_2_ atmosphere. Non-infected BMDMs were stimulated with actinomycin D (Sigma-Aldrich; 2.5 µg/ml) for 6 h as a positive control for apoptosis and with eATP (5 mM) for 24 h as a positive control for necrosis. BMDMs were stained with FITC-labeled annexin V [67] diluted in annexin binding buffer (10 mM HEPES, 150 mM NaCl, 5 mM KCl, 1 mM MgCl_2_, 1.8 mM CaCl_2_) for 20 min and then stained with propidium iodide (PI; Sigma-Aldrich; 1.6 µg/ml). The samples were analyzed in an inverted NIKON TS-100 fluorescence microscope.

### Statistical analysis

Statistical analyses were performed with the GraphPad Prism 4 software (GraphPad, USA), and the differences between the groups were considered significant when *p*<0.05 (5%). The simultaneous effects of two factors were analyzed by two-way ANOVA and the Bonferroni post-hoc test. One-way ANOVA and the Tukey post-hoc test were used to assess the effects of only one parameter. Survival curves were analyzed with the log-rank test of the Kaplan-Meier method.

## Supporting Information

Figure S1 **Kinetics of intracellular bacillus growth and macrophage necrosis in P2X7R^−/−^ and C57BL/6 BMDMs infected with hypervirulent mycobacteria.** C57BL/6 and P2X7R^−/−^ BMDMs were infected with H37Rv *Mtb*, Beijing 1471 *Mtb* and MP287/03 *Mbv* at an MOI of 1 (intracellular bacillus growth) or at an MOI of 20 (macrophage necrosis). (A) On days 0, 3 and 6 p.i., intracellular bacillus growth was assessed by CFU quantification. (B) Macrophage necrosis was evaluated by AO/EthBr incorporation on days 0, 2, 4 and 6 p.i. at an MOI of 20. Significant differences were observed between the C57BL/6 and P2X7R^−/−^ BMDMs (**p<0.01 and ***p<0.001). The data represent the means ± SD of samples in triplicate. The data are representative of three separate experiments.
(TIF)

Figure S2 **Annexin V and PI staining in P2X7R^−/−^ and C57BL/6 BMDMs infected with hypervirulent mycobacteria.** C57BL/6 BMDMs were infected with H37Rv *Mtb*, Beijing 1471 *Mtb* and MP287/03 *Mbv* at an MOI of 10. Non-infected BMDMs were stimulated with actinomycin D (2.5 µg/ml) for 6 h as a positive control for apoptosis and with eATP (5 mM) for 24 h as a positive control for necrosis. Photos show BMDM cultures (200× magnification; bar scales correspond to 100 µm). The data are representative of three separate experiments.
(TIF)

Figure S3 **Extracellular ATP-induced necrosis in C57BL/6 and P2X7R^−/−^ BMDMs.** C57BL/6 and P2X7R^−/−^ BMDMs were cultured for 24 h with 0, 1, 2, 3 and 5 mM eATP. Macrophage necrosis was determined by AO/EthBr incorporation. Images show viable cells (stained with AO) in green and dead cells with permeabilized membranes (stained with EthBr) in red (200× magnification; bar scales correspond to 100 µm). The data are representative of three separate experiments.
(TIF)
