# Supplementary figures and images for: Pulmonary Infection with Hypervirulent Mycobacteria Reveals a Crucial Role for the P2X7 Receptor in Aggressive Forms of Tuberculosis
Source: PLoS Pathog. 2014 Jul 3;10(7):e1004188. doi: 10.1371/journal.ppat.1004188 (PMC4081775; doi:10.1371/journal.ppat.1004188)

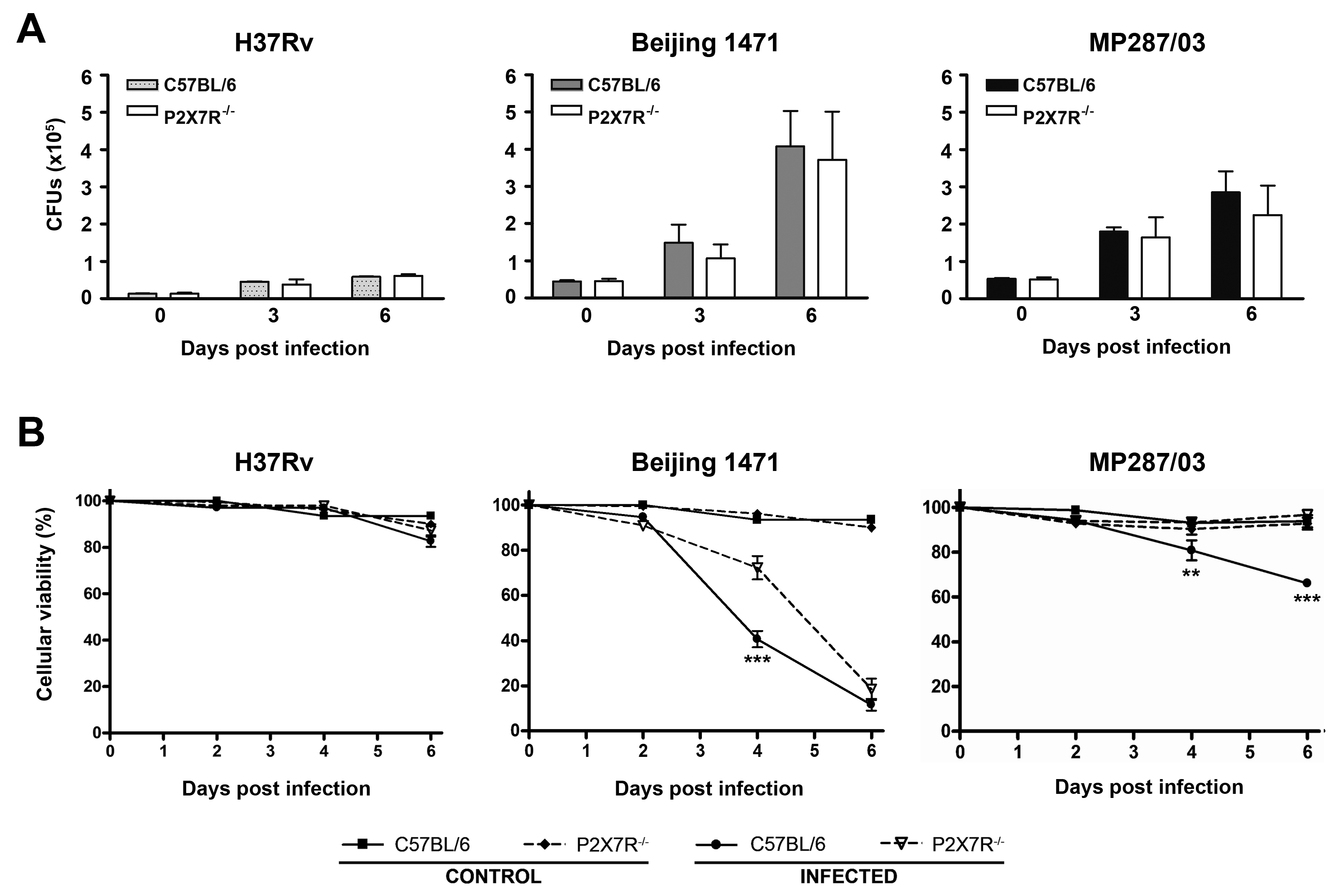

Supplement: Figure S1 — Kinetics of intracellular bacillus growth and macrophage necrosis in P2X7R−/− and C57BL/6 BMDMs infected with hypervirulent mycobacteria. C57BL/6 and P2X7R−/− BMDMs were infected with H37Rv Mtb, Beijing 1471 Mtb and MP287/03 Mbv at an MOI of 1 (intracellular bacillus growth) or at an MOI of 20 (macrophage necrosis). (A) On days 0, 3 and 6 p.i., intracellular bacillus growth was assessed by CFU quantification. (B) Macrophage necrosis was evaluated by AO/EthBr incorporation on days 0, 2, 4 and 6 p.i. at an MOI of 20. Significant differences were observed between the C57BL/6 and P2X7R−/− BMDMs (**p<0.01 and ***p<0.001). The data represent the means ± SD of samples in triplicate. The data are representative of three separate experiments. (TIF) [file ppat.1004188.s001.tif]

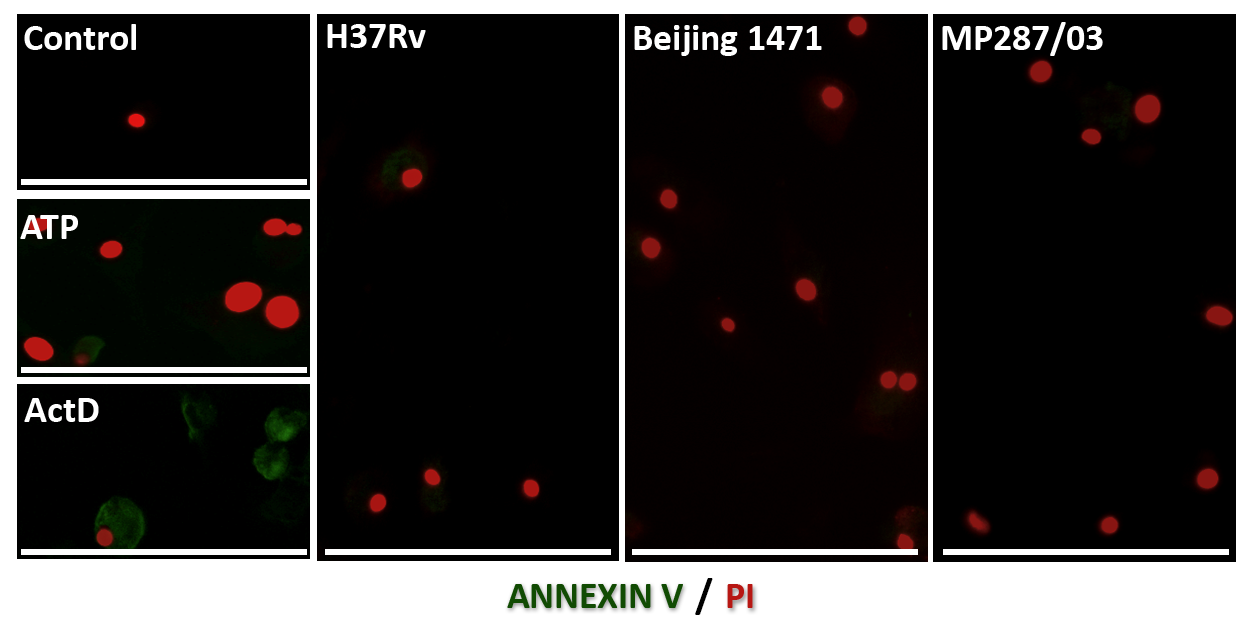

Supplement: Figure S2 — Annexin V and PI staining in P2X7R−/− and C57BL/6 BMDMs infected with hypervirulent mycobacteria. C57BL/6 BMDMs were infected with H37Rv Mtb, Beijing 1471 Mtb and MP287/03 Mbv at an MOI of 10. Non-infected BMDMs were stimulated with actinomycin D (2.5 µg/ml) for 6 h as a positive control for apoptosis and with eATP (5 mM) for 24 h as a positive control for necrosis. Photos show BMDM cultures (200× magnification; bar scales correspond to 100 µm). The data are representative of three separate experiments. (TIF) [file ppat.1004188.s002.tif]

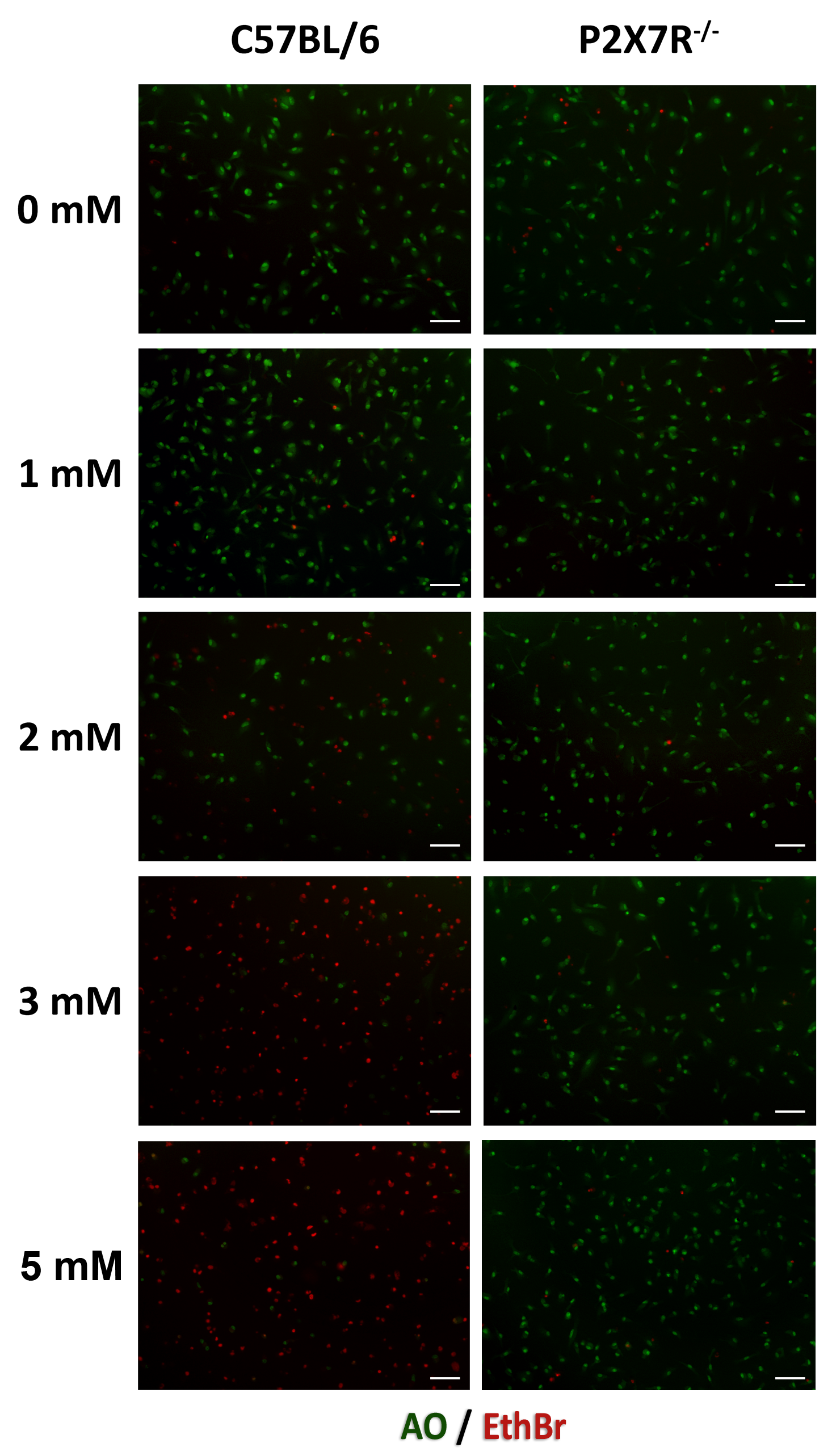

Supplement: Figure S3 — Extracellular ATP-induced necrosis in C57BL/6 and P2X7R−/− BMDMs. C57BL/6 and P2X7R−/− BMDMs were cultured for 24 h with 0, 1, 2, 3 and 5 mM eATP. Macrophage necrosis was determined by AO/EthBr incorporation. Images show viable cells (stained with AO) in green and dead cells with permeabilized membranes (stained with EthBr) in red (200× magnification; bar scales correspond to 100 µm). The data are representative of three separate experiments. (TIF) [file ppat.1004188.s003.tif]
